# Pain intensity and prognosis of acute pancreatitis in an international, prospective study

**DOI:** 10.1093/bjs/znaf155

**Published:** 2025-08-19

**Authors:** Cecilie S Knoph, PAINAP Collaborative, Asbjorn M Drewes, Louise Kuhlmann, Nejo Joseph, John Windsor, Soren Schou Olsen, Chris Varghese, Wei Huang, Jahnvi Dhar, Jayanta Samanta, Rupjyoti Talukdar, Gabriele Capurso, Enrique de-Madaria, Manu Nayar, Matta Kuzman, Neil Cark, James Lucocq, Riinu Pius, Eduardo Houghton, Mariano Gimenéz, Karla Uribe, Florencia Rodriguez, Justin Gundara, Thomas Mackay, Huynh Phan, Joel Lewin, Claire McElhatton, Mehan Siriwardhane, Russell Hodgson, Hassan Malik, Ryan Ward, Kerilee Young, Shaneel Bappayya, Benjamin Loveday, Jaswinder Samra, Tamara Gall, Anubhav Mittal, Ting Ting Chan, Vincent Wing-ho Lo, Hui Liang, Cong Wang, Wei Huang, Tao Jin, Yongzi Wu, Qing Xia, Nikolaou Georgio, Nikolaos Koronakis, Line Davidsen, Emad Hamed, Salem Mohamed, Zaza Demetrashvili, Ana Tvaladze, Irakli Kachakhidze, Tea Zurabashvili, Orestis Ioannidis, Stylianos Kapiris, Eleni Mavrodimitraki, Maria Sotiropoulou, Nikolaos Machairas, Dimitrios Schizas, Athanasios Syllaios, Michail Vailas, Georgios Chlorakis, Evangelos Kalaitzakis, Maria Tsafaridou, Francesk Mulita, Georgios-Ioannis Verras, Amit Gupta, Deepak Rajput, Oshin Sharma, Rajesh Goud, Misbah Unnisa, Lovenish Bains, Nishu Singh, Jahnvi Dhar, Mahmoud Abdelmoeti, Criostóir Ó Súilleabháin, Robert O’Connell, Marcello Calabro, Antonio La Terra, Andrea Muretore, Riccardo Brachet Contul, Margherita Diotallevi, Annamaria Mascaro, Paolo Millo, Santino Antonio Biondo, Carmelo Mazzeo, Eugenio Cucinotta, Francesco Fleres, Aoug Marinak, Veronica Brocco, Marco Ceresoli, Maria Rennis, Danilo Centonze, Coatanza Distefano, Massimiliano Veroux, Domenico Zerbo, Selene Bogoni, Alan Biloslavo, Velentina Bianchi, Marcello Candelli, Francesco Franceschi, Antonio Gasbarrini, Enrico Nista, Gabriele Sganga, Giuseppe Tropeano, Fondazione Policlinico, Caterina Altieri, Vincenza Dinuzzi, Matteo Marconi, Umberto Rivolta, Vitale Roberto Dameno, Mario V Papa, Andrea Balla, Pasquale Lepiane, Federica Saraceno, Alberto Aiolfi, Davide Bona, Andrea Sozzi, Pasquale Cianci, Marco Varesano, Ivana Conversano, Roberta Abete, Raffaele D’Avino, Ester Marra, Gianpaolo Marte, Pasquale Tammaro, Davide Gobatti, Serena Marmaggi, Francesco Palmieri, Roberto Sampietro, Roberto Manca, Federica Pilla, Enrico Piras, Giusto Pignata, Ilaria Canfora, Jacopo Andreuccetti, Rossella D’Alessio, Claudia Armellin, Ugo Grossi, Marco Massani, Alessandro Pontin, Tommaso Stecca, Tiaizna Pilia, Adolfo Pisanu, Mauro Podda, Mario Giuffrida, Gennaro Perrone, Simone Guadagni, Luca Morelli, Alice Frontali, Francesca Basurto, Stefano D’Ugo, Farshad Manoochehri, Marcello Spampinato, Laura Apadula, Paoletta Preatoni, Lodovico Sartarelli, Osama Al-Jaiuossi, Mairam Ernisova, Andrey Sopuev, Bruce Sua, Anthony Farfus, Keith Teo, Brittany Smith, Bathiya Ratnayake, Jayvee Buchanan, Elinor Clark, Saxon Connor, Todd Hore, Salman Attari, Bushra Kadir, Sadik Memon, Zaigham Abbas, Muhammad Ali Quadeer, Abeer Altaf, Pooja Ameet, Jalpa Devi, Nandlal Seerani, Ameer Afzal, Ali Akbar, Mohammad Sohail Asghar, Tiago Sa, Ana Lucia Barreira, Numo Carvalho, Brigitta Cismasiu, Susana Henriques, Francisco Vara Luiz, Andreea Draghici, Valentin Grigorean, Vlad Porojan, Alexandru-Rares Stoian, Lucia Teaca, Dragana Arbutina, Vladica Cuk, Bojan Kovacevic, Luka Mandic, Glenn Bonney, Yujia Gao, Ning Qi Pang, Abdalla Bellil, John Devar, Zafar Khan, Vusi Khumalo, Martin Smith, Sergio Estevez-Fernandez, Beatriz Romero Mosquera, Sergio Rodriguez, Guillermo Garcia-Rayado, Jean Felix Piñerua-Gonsalvez, M Lourdes Ruiz Rebollo, Jose M Olmos, Javier Tejedor-Tejada, Manuel Diez-Alonso, Belen Matias-Garcia, Fernando Mendoza Moreno, Cristina Vera-Mansilla, Helena Salvador Roses, Diego Vázquez Gómez, Juan Rodriguez Oballe, Umesh Jayarajah, Malith Nandasena, Aloka Pathirana, Sami Galal-Eldin, Shahab Hajibandeh, Hytham Hamid, Elif Colak, Larysa Sydorchuk, Ruslan Knut, Ksenia Voronyuk, Serge Chooklin, Vitalii Baryskyi, Ruslan Sydorchuk, Samrat Mukherjee, Maitreyi Patel, Amina Akhtar, Miriam Asarbakhsh, Frances Nolan, Nicholaas Schuijtvlot, Sandhya Prem, Anuradha Thrikandiyur, Millicent Morris, Thomas Mroczek, George Sgourakis, Asma Sultana, Rebecca Varley, Thomas Groot-Wassink, Roland Labinoti, Brett Packham, Keving Seebah, Sophie Allen, Shiva Mokhtassi, Ajay Belgaumkar, Henry De’Ath, Abeerah Pervez, John Mason, Jihene Elkafsi, Amy Cook, Christopher Delaney, Roisin Johnson, Becky Olali Azibaodinami, Ashley Sartini, Mea Stanfield, Ivan Tomasi, Venkat Kanakala, Simon Mbarushimana, Mark McKeever, Mamata Batilli, Gakul Bhatta, Subash Rai, Giles Bond-Smith, Amr Elserafy, Mohamed Shams, Tareq Al Saoudi, Neil Bhardwaj, Wajith Hussain, Francesco Lancellotti, Greta Montagnini, George Cairns, Marianne Hollyman, Asef Rakin, Mishal Shahid, Fraser Barbour, Jake Hawkyard, Matthew McTeer, Sarah Dyer, James Hopkins, Dimitri Pournaras, Alexis Sudlow, S K Kumar, Avinash Aujayeb, Alex Leo, Fatima Lorenzana Senra, Josef Watfah, Javed Latif, Jenifer Barrie, Chris Brown, Dhanny Gomez, Somaiah Aroori, Debora Ciprani, Rahi Karmarkar, Eyas Almomani, Keith Roberts, Madeleine Fale, Ajay Gupta, Max Marsden, Chris Seet, Lakshya Soni, Mohammed Hamdan, Rohan Sadera, Vikas Sud, Edith Chinnah, Davide Di Mauro, Antonio Manzelli, Amira Orabi, Roberto Presa, Alex Reece-Smith, Shahjehan Wajed, Jacob Fingret, Nehal Shah, Jignesh Jatania, Arun Krishna, David Berry, Loukiani Kitsikosta, Jack Helliwell, Benjamin Huntley, James Pine, Jih-Dar Yau, Shiela Lee, Kamal Mahawar, Neehar Shetty, Emily Britton, Alice Shaw, Stijn van Laarhoven, Sukhpreet Gahunia, Miguel Gargia Ortega, Adam Lee, Cho Ee Ng, Jihene El Kafsi, John Mason, Gauri Vithlani, Rami Benhmida, James Gunell, Chetan Parmar, Da-Costa Dorkeh, Mohamed Elnagar, Jih Ian Lee, Ashrafun Nessa, Zhu Hui Yeap, Niroshini Hemadasa, Saria Javed, Sharuk Sami, Dimitrios Damaskos, Andrew Healey, Maria Soupashi, Tania Triantafyllou, Maria Coats, Benjamin Douglass, Brid Hendry, Yasmin Hussain, Zhara Javid, Mia Mantyla, Khaman Rajkumar, Carven Chin, Shahab Hajibandeh, Nagappan Kumar, Ioannis Gerogiannis, Panagiotis Kapsampelis, Farid Gerge, Gulsum Anderson, Vu Dinh, Anna Phillips, Dhiraj Yadav, Sanjay Pandanaboyana

**Affiliations:** Centre for Pancreatic Diseases, Department of Gastroenterology & Hepatology, Aalborg University Hospital, Aalborg, Denmark; Department of Clinical Medicine, Aalborg University, Aalborg, Denmark; HPB and Transplant Unit, Freeman Hospital, Newcastle upon Tyne, UK; Population Health Sciences Institute, Newcastle University, Newcastle Upon Tyne, UK

## Introduction

Acute pancreatitis (AP) is a common inflammatory disease, with increasing incidence in many countries across the world^1–3^. The disease is characterized by abdominal pain, which is often described as severe and intense^4^. The clinical course may vary significantly, ranging from a mild disease that resolves spontaneously to more severe cases. Severe disease can include organ failure and pancreatic necrosis, resulting in significant morbidity and mortality^5^. As such, early identification of patients at risk of severe pancreatitis is crucial for optimizing management and improving outcomes.

Several systems have been used for the early prediction of AP severity, including the Glasgow-Imrie Criteria^6^, Ranson’s Criteria^7^, the Bedside Index of Severity in Acute Pancreatitis score^8^, and the Acute Physiology and Chronic Health Evaluation II score^9^. However, none of these includes pain assessment, despite pain being the presenting symptom. Higher pain intensity may reflect more widespread tissue damage, including pancreatic necrosis and local oedema. As such, specific features of pain, for example abdominal tenderness^10^, shorter duration of pain^11,12^, pain relapse during oral feeding^13^, and increased pain severity^14^, have been associated with severe AP. Given the variability in outcomes of AP, it remains uncertain whether pain intensity alone is sufficient for evaluating pain in AP and whether the assessment of pain intensity would increase the predictive accuracy for the clinical outcomes of AP.

The overall objective of this study was to investigate the relationship between pain intensity and outcomes of AP using prospectively collected data from the PAINAP database^14–16^. The hypothesis was that increased pain intensity on admission would be associated with increased AP severity and risk of complications. The primary aim was to evaluate the association between pain intensity on admission and clinical outcomes of AP. The secondary aim was to determine the predictive accuracy of pain intensity for AP severity and AP-related complications.

## Methods

### Study design

The database used for this post-hoc analysis was from the international, prospective PAINAP study, which has been described in detail in previous publications^14–16^. The study was observational, with the treatment of patients decided solely by the treatment-responsible physicians at each inclusion site. Patients were recruited upon admission within a 3-month interval in 2022, with a 1-month follow-up for each patient. All centres obtained local approval before commencing data collection and the study followed the principles of the Helsinki Declaration. In total, 118 centres across 27 countries participated in the study. All adult patients (≥18 years) admitted directly to inclusion sites with first-time AP were eligible for inclusion. Patients referred from other hospitals were excluded. Other exclusion criteria included chronic pancreatitis, recurrent AP, and pregnancy. For this post-hoc analysis, patients with missing data for key variables (age, sex, pain intensity on admission, or outcomes of AP) were excluded.

### Outcome variables

The PAINAP database included patient demographic details (age, sex, continent of inclusion site, aetiology, and co-morbidity quantified by the Charlson Co-morbidity Index^17^), pain characteristics (duration before admission and intensity on admission), and AP outcomes (severity and local complications according to the revised Atlanta classification^18^, organ failure (including type of organ failure), length of admission reported in days, and mortality). Patients were categorized as having pancreatic necrosis (yes/no) if they developed acute necrotic collections and/or walled-off necrosis. Likewise, patients were categorized as having fluid collections (yes/no) if they developed acute fluid collections and/or pseudocysts. Pain duration before admission was categorized as <12, 12–24, or >24 h. Pain intensity and duration on admission were measured using a numeric rating scale (NRS) of zero to ten, based on interview of the patients or documentation in medical records. Patients were stratified based on pain intensity on admission; they were divided into three categories (no/mild pain (NRS 0–3), moderate pain (NRS 4–6), and severe pain (NRS 7–10)) based on previous reporting and validation of NRS categorization in other conditions^19–22^. Furthermore, patients were categorized based on the use of analgesia before admission (yes/no). This information was registered based on interview and medical files. A representative sample from the PAINAP database was previously re-collected and validated against the original data, demonstrating a moderate to strong correlation, as previously described^14^.

### Statistical analyses

The STATA software packages (StataCorp LP, College Station, Texas, USA; version 17.0) and R (version 4.3) were used for statistical analyses. For patient characteristics, categorical data are presented as *n* (%), whereas continuous data are presented as mean(s.d.) or median (interquartile range (i.q.r.)), depending on the data distribution. Missing data are presented as *n* (%) for each data variable. Before any statistical analyses, patients were categorized into three groups based on pain intensity on admission according to the definitions given above (no/mild pain, moderate pain, or severe pain). To identify outcome variables associated with pain intensity, univariable analyses across these three groups were performed using one-way ANOVA, the Kruskal–Wallis test, Fisher’s exact test, or the chi-squared test, as appropriate according to data type (categorical/continuous) and data distributions. Outcomes associated with pain intensity on univariable analysis were selected for multivariable analysis using binary logistic regression. These multivariable models were adjusted for baseline characteristics that were unevenly distributed between groups (*P* < 0.050), including age, continent, biliary and alcoholic aetiology, and Charlson Co-morbidity Index. Furthermore, the models were adjusted for pre-admission pain duration and the use of analgesia pre-admission based on clinical rationale in accordance with the TRIPOD guidelines^23^. Results are presented as OR (95% c.i.). To assess how pain intensity influenced the probability of clinical outcomes of AP, the area under the curve of the receiver operating characteristics (AUC-ROC), sensitivity, specificity, positive and negative predictive values, prevalence, and likelihood ratios were calculated using a diagnostic accuracy test with outcomes developed during admission (for example disease severity and organ failure) as the reference standard and NRS as the test variable (NRS ≥7 defined as abnormal). The prevalence (∼ pre-test probability) and likelihood ratios were subsequently used to construct nomograms for estimating the post-test probabilities of each outcome assessed. Finally, the multivariable models and probability tests described above were repeated in two subgroup analyses (one analysis excluded patients with pain duration ≤24 before admission and one analysis excluded patients with pain duration >24 h before admission) to assess the robustness of associations across different timings of presentation. *P* < 0.050 was considered statistically significant.

## Results

**Fig. 1:**
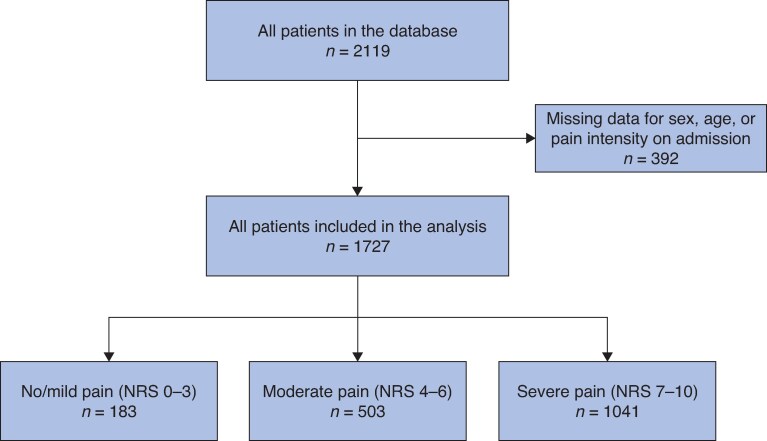
Flow chart for patient inclusion in the study NRS, numeric rating scale.

In total, 1727 patients were included, of whom 183 (11%) reported no/mild pain on admission, 503 (29%) reported moderate pain on admission, and 1041 (60%) reported severe pain on admission (*Fig. 1*). Baseline characteristics stratified according to admission pain intensity are reported in *Table 1*. Of the total population, 810 patients (47%) were female and the median age was 55 (i.q.r. 40–71) years. Biliary aetiology was the most common aetiology (998 patients (58%)). The proportion of patients with alcoholic aetiology was higher for patients with severe pain (253 patients (24%)) and patients with moderate pain (95 patients (19%)) compared with patients with no/mild pain (28 patients (10%)). A Charlson Co-morbidity Index >2 was more frequent for patients with no/mild pain (77 patients (43%)) compared with patients with moderate pain (179 patients (36%)) and patients with severe pain (338 patients (34%)). Finally, most patients had pain for >24 h before admission (799 patients (47%)).

**Table 1:** Baseline characteristics stratified according to pain intensity

	Total population (*n* = 1727)	No/mild pain, NRS 0–3 (*n* = 183)	Moderate pain, NRS 4–6 (*n* = 503)	Severe pain, NRS 7–10 (*n* = 1041)	*P*	Missing data
**Sex**					0.192	0 (0)
Male	917 (53)	99 (54)	250 (50)	568 (55)		
Female	810 (47)	84 (46)	253 (50)	473 (45)		
Age (years), median (i.q.r.)	55 (40–71)	61 (44–76)	56 (41–71)	54 (40–69)	0.003	0 (0)
**Continent**					<0.001	0 (0)
Europe	1177 (68)	101 (55)	356 (71)	720 (69)		
Asia	350 (20)	48 (26)	106 (21)	196 (19)		
Australia	142 (8)	30 (17)	30 (6)	82 (8)		
Other continents*	58 (4)	4 (2)	11 (2)	43 (4)		
Aetiology**						0 (0)
Biliary	998 (58)	109 (60)	312 (62)	577 (55)	0.042	
Alcoholic	366 (21)	28 (10)	95 (19)	253 (24)	<0.001	
Post-ERCP	64 (4)	5 (3)	20 (4)	39 (4)	0.799	
Hypertriglyceridaemia	62 (4)	9 (5)	14 (3)	39 (4)	0.377	
Other	305 (18)	45 (25)	77 (15)	183 (18)	0.019	
**CCI category**					0.003	52 (3)
0	593 (35)	51 (28)	153 (31)	389 (39)		
1–2	488 (29)	53 (29)	162 (33)	273 (27)		
>2	594 (36)	77 (43)	179 (36)	338 (34)		
**Pain duration before admission (h)**					0.480	17 (1)
<12	435 (25)	45 (25)	124 (25)	266 (26)		
12–24	476 (28)	43 (24)	151 (30)	282 (27)		
>24	799 (47)	93 (51)	225 (45)	481 (47)		
**Analgesia pre-admission**					0.141	0 (0)
No	195 (11)	23 (13)	45 (9)	127 (12)		
Yes	1532 (89)	160 (87)	458 (91)	914 (88)		

Values are *n* (%) unless otherwise indicated. *Other continents were South America (*n* = 8), North America (*n* = 24), and Africa (*n* = 26). **Subset of patients had overlapping aetiologies. NRS, numeric rating scale; i.q.r., interquartile range; ERCP, endoscopic retrograde cholangiopancreatography; CCI, Charlson Co-morbidity Index.

### Outcomes associated with pain intensity

On univariable analysis, AP severity according to the revised Atlanta classification^18^ (*P* = 0.002), respiratory organ failure (*P* = 0.028), renal organ failure (*P* < 0.001), pancreatic necrosis (*P* = 0.019), fluid collections (*P* = 0.028), and length of admission (*P* = 0.003) were associated with pain intensity (*Table S1*). The results of the multivariable models are reported in *Table 2*. Severe pain on admission was associated with increased odds of moderately severe/severe AP (OR 1.66 (95% c.i. 1.09 to 2.53); *P* = 0.017), renal organ failure (OR 3.04 (95% c.i. 1.34 to 6.87); *P* = 0.008), and pancreatic necrosis (OR 2.37 (95% c.i. 1.22 to 4.61); *P* = 0.011) compared with no/mild pain. Moderate pain on admission was also associated with increased odds of pancreatic necrosis (OR 2.02 (95% c.i. 1.01 to 4.03); *P* = 0.046) compared with no/mild pain, whereas it was associated with decreased odds of fluid collections (OR 0.56 (95% c.i. 0.33 to 0.96); *P* = 0.035) compared with no/mild pain.

**Table 2:** Multivariable analyses of pain intensity and the odds of developing moderately severe/severe AP, organ failure (any, respiratory, or renal), pancreatic necrosis, or fluid collections (*n* = 1660)

	Adjusted OR (95% c.i.)*	*P*
**Moderately severe/severe AP**		
Moderate pain (*versus* no/mild pain)	1.01 (0.65,1.59)	0.958
Severe pain (*versus* no/mild pain)	1.66 (1.09,2.53)	0.017
**Organ failure**		
Moderate pain (*versus* no/mild pain)	0.69 (0.37,1.27)	0.233
Severe pain (*versus* no/mild pain)	1.79 (1.05,3.08)	0.034
**Respiratory organ failure**		
Moderate pain (*versus* no/mild pain)	0.73 (0.37,1.43)	0.362
Severe pain (*versus* no/mild pain)	1.45 (0.80,2.66)	0.223
**Renal organ failure**		
Moderate pain (*versus* no/mild pain)	0.81 (0.32,2.08)	0.661
Severe pain (*versus* no/mild pain)	3.04 (1.34,6.87)	0.008
**Pancreatic necrosis**		
Moderate pain (*versus* no/mild pain)	2.02 (1.01,4.03)	0.046
Severe pain (*versus* no/mild pain)	2.37 (1.22,4.61)	0.011
**Fluid collections**		
Moderate pain (*versus* no/mild pain)	0.56 (0.33–0.96)	0.035
Severe pain (*versus* no/mild pain)	0.92 (0.57–1.49)	0.725

^*^Adjusted for age, continent, biliary and alcoholic aetiology, Charlson Co-morbidity Index, pre-admission pain duration, and the use of analgesia pre-admission. AP, acute pancreatitis.

### Influence of pain intensity on outcomes

**Fig. 2:**
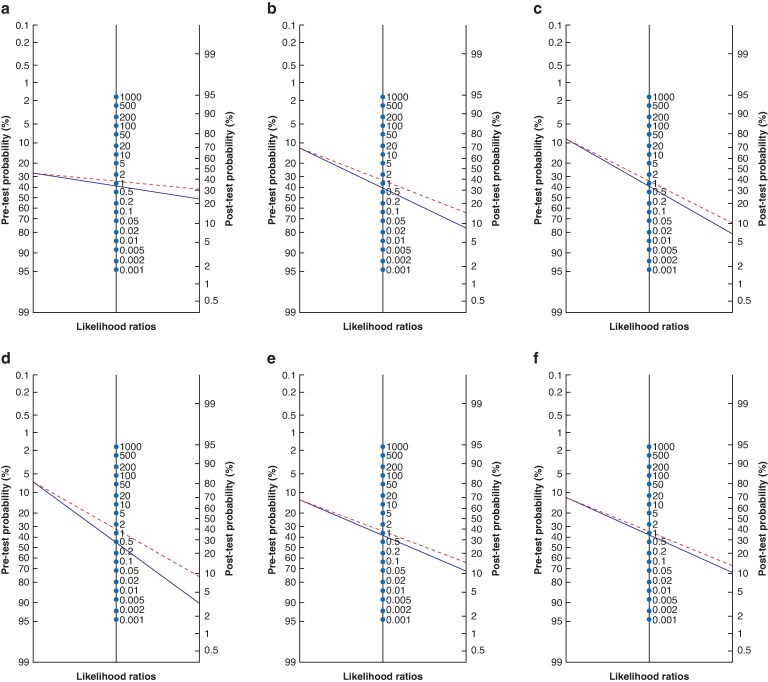
Nomograms of pre- and post-test probabilities and likelihood ratios of an NRS greater than or equal to seven **a** For predicting AP severity according to the revised Atlanta classification. **b** For predicting organ failure. **c** For predicting respiratory organ failure. **d** For predicting renal organ failure. **e** For predicting pancreatic necrosis. **f** For predicting fluid collections. NRS, numeric rating scale; AP, acute pancreatitis.

Overall, the discriminatory ability of severe pain (NRS ≥7) for predicting outcomes of AP was poor, with low to fair AUC-ROCs for AP severity (0.54 (95% c.i. 0.52 to 0.57)), organ failure (0.57 (95% c.i. 0.54 to 0.60)), respiratory organ failure (0.55 (95% c.i. 0.51 to 0.59)), renal organ failure (0.61 (95% c.i. 0.57 to 0.65)), pancreatic necrosis (0.54 (95% c.i. 0.50 to 0.57)), and fluid collections (0.53 (95% c.i. 0.50 to 0.57)) (*Table 3*). The sensitivity was moderate for all outcomes, with the highest value (81% (95% c.i. 72% to 88%)) for renal organ failure. The specificity was lower across all outcomes, ranging from 41% to 42%. Similarly, the positive predictive values were consistently low (9–30%), whereas the negative predictive values were generally higher (77–97%). The highest negative predictive value was observed for renal organ failure. The positive and negative likelihood ratios were poor, indicating that the utilization of pain intensity (cut-off NRS ≥7) did not significantly alter the post-test probability of any outcome compared with the pre-test probability (*Fig. 2*).

**Table 3:** Diagnostic performance of severe baseline pain (NRS 7–10) in diagnosing AP outcomes (*n* = 1727)

Outcome	AUC-ROC (95% c.i.)	Sensitivity (95% c.i.)	Specificity (95% c.i.)	PPV (95% c.i.)	NPV (95% c.i.)
AP severity*	0.54 (0.52,0.57)	67 (62,71)	42 (39,45)	30 (28,33)	77 (74,80)
Organ failure	0.57 (0.54,0.60)	73 (66,79)	41 (39,44)	15 (13,17)	92 (89,94)
Respiratory organ failure	0.55 (0.51,0.59)	69 (61,77)	41 (38,43)	10 (8,12)	93 (91,95)
Renal organ failure	0.61 (0.57,0.65)	81 (72,88)	41 (39,44)	9 (7,11)	97 (95,98)
Pancreatic necrosis	0.54 (0.50,0.57)	67 (60,73)	41 (38,43)	14 (12,17)	89 (87,91)
Fluid collections	0.53 (0.50,0.57)	66 (59,72)	41 (38,43)	13 (11,16)	90 (87–92)

^*^According to the revised Atlanta classification. NRS, numeric rating scale; AP, acute pancreatitis; AUC-ROC, area under the curve of the receiver operating characteristics; PPV, positive predictive value; NPV, negative predictive value.

### Subgroup analyses

The results of the subgroup analyses were consistent with the primary results, except that only renal organ failure and pancreatic necrosis remained significantly associated with severe pain (*Tables S2–S5*).

## Discussion

In this study of patients admitted with first-time AP across a large geographical area, severe pain intensity on admission was associated with more severe AP, renal organ failure, and pancreatic necrosis compared with no/mild pain. Moderate pain intensity on admission was only associated with increased odds of pancreatic necrosis (as well as decreased odds of fluid collections) compared with no/mild pain. However, the discriminatory performance of pain intensity was poor, with low AUC-ROCs and no significant effect on post-test probability for all outcomes. Although pain severity may reflect underlying disease burden in AP, it lacks sufficient accuracy for early risk stratification when used in isolation. However, the high negative predictive values observed in this study suggest that the absence of severe pain on admission may help reassure clinicians and support the decision for outpatient management in low-risk patients.

The pathophysiology of pain in AP is incompletely understood and the majority of the evidence comes from chronic pancreatitis or experimental models of AP. Traditionally, pancreatic pain has been attributed to local inflammation, resulting in tissue damage, ischaemia, oedema, necrosis, and increased ductal pressure^24^. Furthermore, the extensive release of inflammatory mediators during an episode of AP may lead to primary hyperalgesia^25^. This is in contrast to the physiology of pain in painful chronic pancreatitis, for which it is well known that neuropathic pain and central sensitization play an important role^26,27^. While neuropathic pain has yet to be confirmed in AP, neurogenic inflammation, in which injured pancreatic tissue interacts with activated neurons, has been suggested to escalate both pain and inflammation, thus linking severe pain to more severe AP^25,28^. Consistently, an increased risk of organ failure with higher pain intensity was observed in patients with AP. In this context, it is, however, interesting that the association between pain intensity and renal organ failure persisted upon multivariable analysis, whereas the association for respiratory organ failure did not. Acute renal injury during AP may result from increased vascular permeability and hypovolaemia due to systemic inflammation and subsequent renal hypoperfusion, which may be further worsened by abdominal hypertension (and pain)^29,30^. Moreover, factors released from the necrotic pancreas, including activated trypsin, may cause direct nephrotoxicity and impaired renal microcirculation^29,30^. As such, pancreatic necrosis (leading to more severe pain) may increase the risk of acute renal failure to a greater extent than that of acute respiratory dysfunction. Finally, medications such as non-steroidal anti-inflammatory drugs and renin-angiotensin-aldosterone-modulating agents may also affect this.

In the present study, the discriminatory ability of pain intensity for predicting outcomes of AP was poor, indicating that reporting pain intensity is insufficient when it comes to prognostication in AP. Pain intensity is typically measured using an NRS (or a visual analogue scale) due to its simplicity^31^. However, a recent Hungarian study suggested that the descriptive qualities of pain in AP may have a higher prognostic value in predicting disease severity and mortality than pain intensity. For instance, the sharpness of pain sensation was associated with increased severity of AP and mortality^4^. These aspects are, however, rarely assessed in clinical practice and pain intensity is generally an insufficient predictor of disease severity in other acute pain conditions^32–34^. Furthermore, it has been recommended that core outcome sets for acute pain should include at least pain intensity, pain interference, physical function, and quality of life^35^. While many aspects of acute pain are consistent across aetiologies, substantial differences can arise due to anatomical variations in pain origin, as well as differences in pain characteristics influenced by inflammation and neural involvement. Therefore, a more disease-specific approach is warranted. Current pain assessment in AP relies mainly on unidimensional scales, specifically focused on pain intensity. For future pain assessment in AP, a more comprehensive multidimensioned tool for pain assessment, as with chronic pancreatitis, may be warranted^36^. The ongoing CAPPOS study undertaken by the authors’ group aims to create core outcome sets specifically for pain in AP, including all relevant disease-related aspects of pain, and develop assessment tools for each outcome^37^. This approach may enable a more precise evaluation of the relationship between pain, disease severity, and prognosis.

The primary strength of the present study was the multicentred, prospective, international design, including the large study cohort, enhancing generalizability and the completeness of data, which were validated after initial data collection. Pain intensity was self-reported and, therefore, subject to significant inter-individual variation, as the pain experience is subjective and can vary considerably between individuals. The analyses did not account for pre-admission analgesia. However, as all patients were directly admitted to one of the inclusion centres and not transferred from other centres, any pre-admission analgesia would have been self-administered and the use of strong analgesics before admission was likely minimal. Another limitation was the snapshot design of the database, which only recorded pain intensity on admission. Repeated pain assessments over time would have allowed for an analysis of its temporal evolution, potentially offering further insight into the relationship between pain and prognosis. In this study, strong associations were found between severe pain on admission and several clinical outcomes of AP. However, the discriminatory performance of severe pain in predicting these outcomes was poor. This is likely because some patients recover spontaneously, despite experiencing severe pain on presentation. Nevertheless, the higher negative predictive values suggest that the absence of severe pain on admission may help rule out certain adverse outcomes, particularly renal failure.

The present study showed that severe pain was associated with worse outcomes of AP, but the discriminatory performance of using severe pain to predict outcomes of AP was poor. Future research is warranted to refine the assessment of AP pain and clarify the underlying mechanisms. A more comprehensive understanding of pain in AP could improve its prognostic value, guide more personalized treatment strategies, and optimize pain management. This, in turn, may aid in monitoring treatment response, informing analgesic dose adjustments, and determining the timing of different analgesic strategies, ultimately improving both clinical and patient-centred outcomes.

## Collaborators

Asbjorn M. Drewes (Aalborg University, Denmark); Louise Kuhlmann (Aalborg University, Denmark); Nejo Joseph (Auckland City Hospital, Auckland, New Zealand); John Windsor (Auckland City Hospital, Auckland, New Zealand); Soren Schou Olsen (Aalborg University, Denmark); Chris Varghese (Auckland City Hospital, Auckland, New Zealand); Wei Huang (West China Hospital, Sichuan University, China); Jahnvi Dhar (PGI Institute, Chandigarh, India); Jayanta Samanta (PGI Institute, Chandigarh, India); Rupjyoti Talukdar (AIG Hospitals, Hyderabad); Gabriele Capurso (Vita-Salute San Raffaele University); Enrique de-Madaria (Dr Balmis General University Hospital, Alicante, Spain); Manu Nayar (Freeman Hospital, Newcastle, UK); Matta Kuzman (Freeman Hospital, Newcastle, UK); Neil Cark (Data Manager, University of Edinburgh, UK); James Lucocq (Royal Infirmary, Edinburgh, UK); Riinu Pius (Senior Data Manager in Surgical Informatics, University of Edinburgh, UK); Eduardo Houghton (Rivadavia Hospital, Buenos Aires); Mariano Gimenéz (Rivadavia Hospital, Buenos Aires); Karla Uribe (Rivadavia Hospital, Buenos Aires); Florencia Rodriguez (Rivadavia Hospital, Buenos Aires); Justin Gundara (Logan Hospital, Queensland, Australia); Thomas Mackay (Logan Hospital, Queensland, Australia); Huynh Phan (Logan Hospital, Queensland, Australia); Joel Lewin (Mater Hospital, Sydney, Australia); Claire McElhatton (Mater Hospital, Sydney, Australia); Mehan Siriwardhane (Mater Hospital, Sydney, Australia); Russell Hodgson (Northern Health Hospital, Melbourne, Australia); Hassan Malik (Northern Health Hospital, Melbourne, Australia); Ryan Ward (Northern Health Hospital, Melbourne, Australia); Kerilee Young (Northern Health Hospital, Melbourne, Australia); Shaneel Bappayya (Melbourne Hospital, Melbourne, Australia); Benjamin Loveday (Royal Melbourne Hospital, Melbourne, Australia); Jaswinder Samra (Royal North Shore Hospital, Sydney, Australia); Tamara Gall (Royal North Shore Hospital, Sydney, Australia); Anubhav Mittal (Royal North Shore Hospital, Sydney, Australia); Ting Ting Chan (Prince of Wales Hospital, Sha Tin, Hong Kong); Vincent Wing-ho Lo (Prince of Wales Hospital, Sha Tin, Hong Kong); Hui Liang (Second Affiliated Hospital of Nanchang University, Jiangxi, China); Cong Wang (Second Affiliated Hospital of Nanchang University, Jiangxi, China); Wei Huang (West China Hospital of Sichuan University, Chengdu, Sichuan, China); Tao Jin (West China Hospital of Sichuan University, Chengdu, Sichuan, China); Yongzi Wu (West China Hospital of Sichuan University, Chengdu, Sichuan, China); Qing Xia (West China Hospital of Sichuan University, Chengdu, Sichuan, China); Nikolaou Georgio (Larnaca General Hospital, Larnaca, Cyprus); Nikolaos Koronakis (Larnaca General Hospital, Larnaca, Cyprus); Line Davidsen (Aalborg University Hospital, Aalborg, Denmark); Emad Hamed (Zagzig University, Zagazig, Egypt); Salem Mohamed (Zagzig University, Zagazig, Egypt); Zaza Demetrashvili (Tbilisi State Medical University, Tbilisi, Georgia); Ana Tvaladze (Tbilisi State Medical University, Tbilisi, Georgia); Irakli Kachakhidze (Tbilisi State Medical University, Tbilisi, Georgia); Tea Zurabashvili (Tbilisi State Medical University, Tbilisi, Georgia); Orestis Ioannidis (Aristotle University of Thessaloniki, Thessaloniki, Greece); Stylianos Kapiris (Evaggelismos General Hospital, Athens, Greece); Eleni Mavrodimitraki (Evaggelismos General Hospital, Athens, Greece); Maria Sotiropoulou (Evaggelismos General Hospital, Athens, Greece); Nikolaos Machairas (Laikon General Hospital, Athens, Greece); Dimitrios Schizas (Laikon General Hospital, Athens, Greece); Athanasios Syllaios (Laikon General Hospital, Athens, Greece); Michail Vailas (Laikon General Hospital, Athens, Greece); Georgios Chlorakis (University Hospital of Heraklion, Iraklio, Greece); Evangelos Kalaitzakis (University Hospital of Heraklion, Iraklio, Greece); Maria Tsafaridou (University Hospital of Heraklion, Iraklio, Greece); Francesk Mulita (University of Patras,Patras, Greece); Georgios-Ioannis Verras (University of Patras, Patras, Greece); Amit Gupta (All India Institute of Medical Sciences, New Delhi, India); Deepak Rajput (All India Institute of Medical Sciences, New Delhi, India); Oshin Sharma (All India Institute of Medical Sciences, New Delhi, India); Rajesh Goud (Hyderabad, India); Misbah Unnisa (Hyderabad, India); Lovenish Bains (Maulana Azad Medical College & Lok Nayak Hospital, New Delhi, India); Nishu Singh (Maulana Azad Medical College & Lok Nayak Hospital, New Delhi, India); Jahnvi Dhar (Post Graduate Institute of Medical Education and Research Chandigarh, Chandigarh, India); Mahmoud Abdelmoeti (Mercy University Hospital, Cork, Ireland); Criostóir Ó Súilleabháin (Mercy University Hospital, Cork, Ireland); Robert O’Connell (Mercy University Hospital, Cork, Ireland); Marcello Calabro (Agnelli Hospital, Pinerolo, Italy); Antonio La Terra (Agnelli Hospital, Pinerolo, Italy); Andrea Muretore (Agnelli Hospital, Pinerolo, Italy); Riccardo Brachet Contul (Hôpital régional Umberto Parini, Aosta, Italy); Margherita Diotallevi (Hôpital régional Umberto Parini, Aosta, Italy); Annamaria Mascaro (Hôpital régional Umberto Parini, Aosta, Italy); Paolo Millo (Hôpital régional Umberto Parini, Aosta, Italy); Santino Antonio Biondo (Policlinic of Messina, Sicily, Italy); Carmelo Mazzeo (Policlinic of Messina, Sicily, Italy); Eugenio Cucinotta (Policlinic of Messina, Sicily, Italy); Francesco Fleres (Policlinic of Messina, Sicily, Italy); Aoug Marinak (Policlinic of Messina, Sicily, Italy); Veronica Brocco (ASST Monza—San Gerado Hospital, Monza, Italy); Marco Ceresoli (ASST Monza—San Gerado Hospital, Monza, Italy); Maria Rennis (ASST Monza—San Gerado Hospital, Monza, Italy); Danilo Centonze (Azioenda Policlinico San Marco, Catania, Italy); Coatanza Distefano (Azioenda Policlinico San Marco, Catania, Italy); Massimiliano Veroux (Azioenda Policlinico San Marco, Catania, Italy); Domenico Zerbo (Azioenda Policlinico San Marco, Catania, Italy); Selene Bogoni (Cattinara University Hospital, Cattinara, Italy); Alan Biloslavo (Cattinara University Hospital, Cattinara, Italy); Velentina Bianchi (Fondazione Policlinico Universitario Agostino gemelli IRCCS, Rome, Italy); Marcello Candelli (Fondazione Policlinico Universitario Agostino gemelli IRCCS, Rome, Italy); Francesco Franceschi (Fondazione Policlinico Universitario Agostino gemelli IRCCS, Rome, Italy); Antonio Gasbarrini (Fondazione Policlinico Universitario Agostino gemelli IRCCS, Rome, Italy); Enrico Nista (Fondazione Policlinico Universitario Agostino gemelli IRCCS, Rome, Italy); Gabriele Sganga (Fondazione Policlinico Universitario Agostino gemelli IRCCS, Rome, Italy); Giuseppe Tropeano (Fondazione Policlinico Universitario Agostino gemelli IRCCS, Rome, Italy); Fondazione Policlinico (Universitario Agostino gemelli IRCCS, Rome, Italy); Caterina Altieri (Giuseppe Fornaroli Hospital, Magenta, Italy); Vincenza Dinuzzi (Giuseppe Fornaroli Hospital, Magenta, Italy); Matteo Marconi (Giuseppe Fornaroli Hospital, Magenta, Italy); Umberto Rivolta (Giuseppe Fornaroli Hospital, Magenta, Italy); Vitale Roberto Dameno (Giuseppe Fornaroli Hospital, Magenta, Italy); Mario V. Papa (Hospital of Caserta, Caserta, Italy); Andrea Balla (Hospital San Paolo, Santana, Italy); Pasquale Lepiane (Hospital San Paolo, Santana, Italy); Federica Saraceno (Hospital San Paolo, Santana, Italy); Alberto Aiolfi (Istuto Clinico Sant’ Ambrogio, Milan, Italy); Davide Bona (Istuto Clinico Sant’ Ambrogio, Milan, Italy); Andrea Sozzi (Istuto Clinico Sant’ Ambrogio, Milan, Italy); Pasquale Cianci (Lorenzo Bonomo Hospital, Andria, Italy); Marco Varesano (Lorenzo Bonomo Hospital, Andria, Italy); Ivana Conversano (Lorenzo Bonomo Hospital, Andria, Italy); Roberta Abete (Ospedale Del Mare, Naples, Italy); Raffaele D’Avino (Ospedale Del Mare, Naples, Italy); Ester Marra (Ospedale Del Mare, Naples, Italy); Gianpaolo Marte (Ospedale Del Mare, Naples, Italy); Pasquale Tammaro (Andrea Tufo, Ospedale Del Mare, Naples, Italy); Davide Gobatti (Ospedale Moriggia Pelascini, Gravedona, Italy); Serena Marmaggi (Ospedale Moriggia Pelascini, Gravedona, Italy); Francesco Palmieri (Ospedale Moriggia Pelascini, Gravedona, Italy); Roberto Sampietro (Ospedale Moriggia Pelascini, Gravedona, Italy); Roberto Manca (Ospedale Santissima Trinità, Cagliari, Italy); Federica Pilla (Ospedale Santissima Trinità, Cagliari, Italy); Enrico Piras (Ospedale Santissima Trinità, Cagliari, Italy); Giusto Pignata (ASST Spedali Civili of Brescia, Brescia, Italy); Ilaria Canfora (ASST Spedali Civili of Brescia, Brescia, Italy); Jacopo Andreuccetti (ASST Spedali Civili of Brescia, Brescia, Italy); Rossella D’Alessio (ASST Spedali Civili of Brescia, Brescia, Italy); Claudia Armellin (Treviso Regional Hospital, Treviso, Italy); Ugo Grossi (Treviso Regional Hospital, Treviso, Italy); Marco Massani (Treviso Regional Hospital, Treviso, Italy); Alessandro Pontin (Treviso Regional Hospital, Treviso, Italy); Tommaso Stecca (Treviso Regional Hospital, Treviso, Italy); Tiaizna Pilia (University of Cagliari, Cagliari, Italy); Adolfo Pisanu (University of Cagliari, Cagliari, Italy); Mauro Podda (University of Cagliari, Cagliari, Italy); Mario Giuffrida (University of Parma, Parma, Italy); Gennaro Perrone (University of Parma, Parma, Italy); Simone Guadagni (University of Pisa, Pisa, Italy); Luca Morelli (University of Pisa, Pisa, Italy); Alice Frontali (Vimercate Hospital, Vimercate, Italy); Francesca Basurto (Vito Fazzi Hospital, Lecce, Italy); Stefano D’Ugo (Vito Fazzi Hospital, Lecce, Italy); Farshad Manoochehri (Vito Fazzi Hospital, Lecce, Italy); Marcello Spampinato (Vito Fazzi Hospital, Lecce, Italy); Laura Apadula (San Raffaele institute, Milan, Italy); Paoletta Preatoni (San Raffaele institute, Milan, Italy); Lodovico Sartarelli (SS. Annunziata Hospital, Taranto, Italy); Osama Al-Jaiuossi (AlBashir Hospital, Amman, Jordan); Mairam Ernisova (M. M. Mamakeev, Bishkek, Kyrgyzstan); Andrey Sopuev (M. M. Mamakeev, Bishkek, Kyrgyzstan); Bruce Sua (Auckland City Hospital, Auckland, New Zealand); Anthony Farfus (Auckland City Hospital, Auckland, New Zealand); Keith Teo (Auckland City Hospital, Auckland, New Zealand); Brittany Smith (Auckland City Hospital, Auckland, New Zealand); Bathiya Ratnayake (Auckland City Hospital, Auckland, New Zealand); Jayvee Buchanan (Canterbury District Health Board, Canterbury, New Zealand); Elinor Clark (Canterbury District Health Board, Canterbury, New Zealand); Saxon Connor (Canterbury District Health Board, Canterbury, New Zealand); Todd Hore (Canterbury District Health Board, Canterbury, New Zealand); Salman Attari (Asian Institute of Medical Sciences (AIMS) Hospital, Hyderabad, Pakistan); Bushra Kadir (Asian Institute of Medical Sciences (AIMS) Hospital, Hyderabad, Pakistan); Sadik Memon (Asian Institute of Medical Sciences (AIMS) Hospital, Hyderabad, Pakistan); Zaigham Abbas (Dr Ziauddin University Hospital, Sindh, Pakistan); Muhammad Ali Quadeer (Dr Ziauddin University Hospital, Sindh (Pakistan); Abeer Altaf (Dr Ziauddin University Hospital, Sindh, Pakistan); Pooja Ameet (Liquiat University of Medical and Health Sciences, Sindh, Pakistan); Jalpa Devi (Liquiat University of Medical and Health Sciences, Sindh, Pakistan); Nandlal Seerani (Liquiat University of Medical and Health Sciences, Sindh, Pakistan); Ameer Afzal (Mayo Hospital, Lahore, Pakistan); Ali Akbar (Mayo Hospital, Lahore, Pakistan); Mohammad Sohail Asghar (Mayo Hospital, Lahore, Pakistan); Tiago Sa (Centro Hospitalar do Tamege e Sousa, Guilhufe, Portugal); Ana Lucia Barreira (Hospital Garcia de Orta, Almada, Portugal); Numo Carvalho (Hospital Garcia de Orta, Almada, Portugal); Brigitta Cismasiu (Hospital Garcia de Orta, Almada, Portugal); Susana Henriques (Hospital Garcia de Orta, Almada, Portugal); Francisco Vara Luiz (Hospital Garcia de Orta, Almada, Portugal); Andreea Draghici (Bagdasar Arseni Emergency Hospital, Bucharest, Romania); Valentin Grigorean (Bagdasar Arseni Emergency Hospital, Bucharest, Romania); Vlad Porojan (Bagdasar Arseni Emergency Hospital, Bucharest, Romania); Alexandru-Rares Stoian (Bagdasar Arseni Emergency Hospital, Bucharest, Romania); Lucia Teaca (Bagdasar Arseni Emergency Hospital, Bucharest, Romania); Dragana Arbutina (UMC Zvezdara, Zvezdara, Serbia); Vladica Cuk (UMC Zvezdara, Zvezdara, Serbia); Bojan Kovacevic (UMC Zvezdara, Zvezdara, Serbia); Luka Mandic (UMC Zvezdara, Zvezdara, Serbia); Glenn Bonney (National University Hospital, Singapore); Yujia Gao (National University Hospital, Singapore); Ning Qi Pang (National University Hospital, Singapore); Abdalla Bellil (Chris Hani Baragwanath Academic Hospital, Johannesburg, South Africa); John Devar (Chris Hani Baragwanath Academic Hospital, Johannesburg, South Africa); Zafar Khan (Chris Hani Baragwanath Academic Hospital, Johannesburg, South Africa); Vusi Khumalo (Chris Hani Baragwanath Academic Hospital, Johannesburg, South Africa); Martin Smith (Chris Hani Baragwanath Academic Hospital, Johannesburg, South Africa); Sergio Estevez-Fernandez (Complejo Hospitalario Universitario de Vigo, Vigo, Spain); Beatriz Romero Mosquera (Complejo Hospitalario Universitario de Vigo, Vigo, Spain); Sergio Rodriguez (Complejo Hospitalario Universitario de Vigo, Vigo, Spain); Guillermo Garcia-Rayado (Hospital Clínico Universitario Lozano Blesa, Zaragoza, Spain); Jean Felix Piñerua-Gonsalvez (Hospital Clinico Universitario Valladolid, Valladolid, Spain); M. Lourdes Ruiz Rebollo (Hospital Clinico Universitario Valladolid, Valladolid, Spain); Jose M. Olmos (Hospital Universitario de Cabuenes, Asturias, Spain); Javier Tejedor-Tejada (Hospital Universitario de Cabuenes, Asturias, Spain); Manuel Diez-Alonso (Hosptial Universitation Principe de Asturias, Alcalá de Henares, Spain); Belen Matias-Garcia (Hosptial Universitation Principe de Asturias, Alcalá de Henares, Spain); Fernando Mendoza Moreno (Hosptial Universitation Principe de Asturias, Alcalá de Henares, Spain); Cristina Vera-Mansilla (Hosptial Universitation Principe de Asturias, Alcalá de Henares, Spain); Helena Salvador Roses (University Hospital Arnau de Vilanova, Lleida, Spain); Diego Vázquez Gómez (University Hospital Arnau de Vilanova, Lleida, Spain); Juan Rodriguez Oballe (University Hospital Arnau de Vilanova, Lleida, Spain); Umesh Jayarajah (Colombo South Teaching Hospital, Dehiwala-Mount Lavinia, Sri lanka); Malith Nandasena (Colombo South Teaching Hospital, Dehiwala-Mount Lavinia, Sri lanka); Aloka Pathirana (Colombo South Teaching Hospital, Dehiwala-Mount Lavinia, Sri lanka); Sami Galal-Eldin (Al-Maolem Medical City, Khartoum, Sudan); Shahab Hajibandeh (Al-Maolem Medical City, Khartoum, Sudan); Hytham Hamid (Al-Maolem Medical City, Khartoum, Sudan); Elif Colak (Samsun Training and Research Hospital, Samsun, Turkey); Larysa Sydorchuk (Bukovinian State Medical University, Chernivtsi, Ukraine); Ruslan Knut (Bukovinian State Medical University, Chernivtsi, Ukraine); Ksenia Voronyuk (Bukovinian State Medical University, Chernivtsi, Ukraine); Serge Chooklin (Lviv Regional Clinical Hospital, Lviv, Ukraine); Vitalii Baryskyi (Chernivtsi Regional Emergency Hospital, Chernivtsi, Ukraine); Ruslan Sydorchuk (Chernivtsi Regional Emergency Hospital, Chernivtsi, Ukraine); Samrat Mukherjee (Barking, Havering and Redbridge University Hospitals, Ramford, UK); Maitreyi Patel (Barking, Havering and Redbridge University Hospitals, Ramford, UK); Amina Akhtar (Chesterfield Royal Hospital, Chesterfield, UK); Miriam Asarbakhsh (Chesterfield Royal Hospital, Chesterfield, UK); Frances Nolan (Chesterfield Royal Hospital, Chesterfield, UK); Nicholaas Schuijtvlot (Chesterfield Royal Hospital, Chesterfield, UK); Sandhya Prem (Darlington Memorial Hospital, Darlington UK); Anuradha Thrikandiyur (Darlington Memorial Hospital, Darlington UK); Millicent Morris (East Lancashire Hospitals NHS Trust, Lancashire, UK); Thomas Mroczek (East Lancashire Hospitals NHS Trust, Lancashire, UK); George Sgourakis (East Lancashire Hospitals NHS Trust, Lancashire, UK); Asma Sultana (East Lancashire Hospitals NHS Trust, Lancashire, UK); Rebecca Varley (East Lancashire Hospitals NHS Trust, Lancashire, UK); Thomas Groot-Wassink (East Suffock and North Essex Trust, Colchester, Essex, UK); Roland Labinoti (East Suffock and North Essex Trust, Colchester, Essex, UK); Brett Packham (East Suffock and North Essex Trust, Colchester, Essex, UK); Keving Seebah (East Suffock and North Essex Trust, Colchester, Essex, UK); Sophie Allen (East Surrey Hospital, Surrey, UK); Shiva Mokhtassi (East Surrey Hospital, Surrey, UK); Ajay Belgaumkar (East Surrey Hospital, Surrey, UK); Henry De’Ath (Frimley Park Hospital, London, UK); Abeerah Pervez (Frimley Park Hospital, London, UK); John Mason (Frimley Park Hospital, London, UK); Jihene Elkafsi (Frimley Park Hospital, London, UK); Amy Cook (Guys and St Thomas Hospital, London, UK); Christopher Delaney (Guys and St Thomas Hospital, London, UK); Roisin Johnson (Guys and St Thomas Hospital, London, UK); Becky Olali Azibaodinami (Guys and St Thomas Hospital, London, UK); Ashley Sartini (Guys and St Thomas Hospital, London, UK); Mea Stanfield (Guys and St Thomas Hospital, London, UK); Ivan Tomasi (Guys and St Thomas Hospital, London, UK); Venkat Kanakala (James Cook University Hospital, Middlesbrough, UK); Simon Mbarushimana (James Cook University Hospital, Middlesbrough, UK); Mark McKeever (James Cook University Hospital, Middlesbrough, UK); Mamata Batilli (James Paget NHS Trust, Norfolk, UK); Gakul Bhatta (James Paget NHS Trust, Norfolk, UK); Subash Rai (James Paget NHS Trust, Norfolk, UK); Giles Bond-Smith (John Radcliffe Hospital, Oxford, UK); Amr Elserafy (John Radcliffe Hospital, Oxford, UK); Mohamed Shams (John Radcliffe Hospital, Oxford, UK); Tareq Al Saoudi (Leicester General Hospital, Leicester, UK); Neil Bhardwaj (Leicester General Hospital, Leicester, UK); Wajith Hussain (Leicester General Hospital, Leicester, UK); Francesco Lancellotti (Manchester Royal Infirmary, Manchester, UK); Greta Montagnini (Manchester Royal Infirmary, Manchester, UK); George Cairns (Musgrove Park Hospital, Somerset, UK); Marianne Hollyman (Musgrove Park Hospital, Somerset, UK); Asef Rakin (Musgrove Park Hospital, Somerset, UK); Mishal Shahid (Musgrove Park Hospital, Somerset, UK); Fraser Barbour (Freeman Hospital, Newcastle upon Tyne, UK); Jake Hawkyard (Freeman Hospital, Newcastle upon Tyne, UK); Matthew McTeer (Freeman Hospital, Newcastle upon Tyne, UK); Sarah Dyer (North Bristol NHS Trust, Bristol, UK); James Hopkins (North Bristol NHS Trust, Bristol, UK); Dimitri Pournaras (North Bristol NHS Trust, Bristol, UK); Alexis Sudlow (North Bristol NHS Trust, Bristol, UK); S. K. Kumar (Northumbria NHS Foundation Trust, Northumbria, UK); Avinash Aujayeb (Northumbria NHS Foundation Trust, Northumbria, UK); Alex Leo (Northwick Park Hospital, Watford, UK); Fatima Lorenzana Senra (Northwick Park Hospital, Watford, UK); Josef Watfah (Northwick Park Hospital, Watford, UK); Javed Latif (Nottingham University Hospitals, Nottingham, UK); Jenifer Barrie (Nottingham University Hospitals, Nottingham, UK); Chris Brown (Nottingham University Hospitals, Nottingham, UK); Dhanny Gomez (Nottingham University Hospitals, Nottingham, UK); Somaiah Aroori (Plymouth Hospital NHS Trust, Plymouth, UK); Debora Ciprani (Plymouth Hospital NHS Trust, Plymouth, UK); Rahi Karmarkar (Plymouth Hospital NHS Trust, Plymouth, UK); Eyas Almomani (Queen Elizabeth Hospital, Birmingham, UK); Keith Roberts (Queen Elizabeth Hospital, Birmingham, UK); Madeleine Fale (Queen Elizabeth Hospital, Gateshead, UK); Ajay Gupta (Queen Elizabeth Hospital, Gateshead, UK); Max Marsden (Queen Elizabeth Hospital, London, UK); Chris Seet (Queen Elizabeth Hospital, London, UK); Lakshya Soni (Queen Elizabeth Hospital, London, UK); Mohammed Hamdan (Royal Berkshire Hospital, Reading, UK); Rohan Sadera (Royal Berkshire Hospital, Reading, UK); Vikas Sud (Royal Berkshire Hospital, Reading, UK); Edith Chinnah (Royal Devon University Healthcare Foundation Trust, Devon, UK); Davide Di Mauro (Royal Devon University Healthcare Foundation Trust, Devon, UK); Antonio Manzelli (Royal Devon University Healthcare Foundation Trust, Devon, UK); Amira Orabi (Royal Devon University Healthcare Foundation Trust, Devon, UK); Roberto Presa (Royal Devon University Healthcare Foundation Trust, Devon, UK); Alex Reece-Smith (Royal Devon University Healthcare Foundation Trust, Devon, UK); Shahjehan Wajed (Royal Devon University Healthcare Foundation Trust, Devon, UK); Jacob Fingret (Sheffield Teaching Hospital, Sheffield, UK); Nehal Shah (Sheffield Teaching Hospital, Sheffield, UK); Jignesh Jatania (South Tyneside District Hospital, South Sheilds, UK); Arun Krishna (South Tyneside District Hospital, South Sheilds, UK); David Berry (University Hospital Southampton NHS Foundation Trust, Southampton, UK); Loukiani Kitsikosta (University Hospital Southampton NHS Foundation Trust, Southampton, UK); Jack Helliwell (St James’s University Hospital, Leeds, UK); Benjamin Huntley (St James’s University Hospital, Leeds, UK); James Pine (St James’s University Hospital, Leeds, UK); Jih-Dar Yau (St James’s University Hospital, Leeds, UK); Shiela Lee (Sunderland Royal Hospital, Sunderland, UK); Kamal Mahawar (Sunderland Royal Hospital, Sunderland, UK); Neehar Shetty (Sunderland Royal Hospital, Sunderland, UK); Emily Britton (University Hospitals Bristol & Weston NHS Foundation Trust, Bristol, UK); Alice Shaw (University Hospitals Bristol & Weston NHS Foundation Trust, Bristol, UK); Stijn van Laarhoven (University Hospitals Bristol & Weston NHS Foundation Trust, Bristol, UK); Sukhpreet Gahunia (University Hospital Lewisham, Lewisham, UK); Miguel Gargia Ortega (University Hospital of North Durham, Durham, UK); Adam Lee (University Hospital of North Durham, Durham, UK); Cho Ee Ng (University Hospital of North Durham, Durham, UK); Jihene El Kafsi (Wexham Park Hospital, Slough, UK); John Mason (Wexham Park Hospital, Slough, UK); Gauri Vithlani (Wexham Park Hospital, Slough, UK); Rami Benhmida (Whittington Health NHS Trust, London, UK); James Gunell (Whittington Health NHS Trust, London, UK); Chetan Parmar (Whittington Health NHS Trust, London, UK); Da-Costa Dorkeh (Aberdeen Royal Infirmary, Aberdeen, UK); Mohamed Elnagar (Aberdeen Royal Infirmary, Aberdeen, UK); Jih Ian Lee (Aberdeen Royal Infirmary, Aberdeen, UK); Ashrafun Nessa (Aberdeen Royal Infirmary, Aberdeen, UK); Zhu Hui Yeap (Aberdeen Royal Infirmary, Aberdeen, UK); Niroshini Hemadasa (Dumfries and Galloway Royal Infirmary, Dumfries, UK); Saria Javed (Dumfries and Galloway Royal Infirmary, Dumfries, UK); Sharuk Sami (Dumfries and Galloway Royal Infirmary, Dumfries, UK); Dimitrios Damaskos (Royal Infirmary of Edinburgh, UK); Andrew Healey (Royal Infirmary of Edinburgh, UK); Maria Soupashi (Royal Infirmary of Edinburgh, UK); Tania Triantafyllou (Royal Infirmary of Edinburgh, UK); Maria Coats (Glasgow Royal Infirmary, Glasgow, UK); Benjamin Douglass (Glasgow Royal Infirmary, Glasgow, UK); Brid Hendry (Glasgow Royal Infirmary, Glasgow, UK); Yasmin Hussain (Glasgow Royal Infirmary, Glasgow, UK); Zhara Javid (Glasgow Royal Infirmary, Glasgow, UK); Mia Mantyla (Glasgow Royal Infirmary, Glasgow, UK); Khaman Rajkumar (Glasgow Royal Infirmary, Glasgow, UK); Carven Chin (Cardiff and Vale UHB, Cardiff, UK); Shahab Hajibandeh (Cardiff and Vale UHB, Cardiff, UK); Nagappan Kumar (Cardiff and Vale UHB, Cardiff, UK); Ioannis Gerogiannis (Kingston Hospital NHS Foundation Trust, Kingston-upon-Thames, UK); Panagiotis Kapsampelis (Kingston Hospital NHS Foundation Trust, Kingston-upon-Thames, UK); Farid Gerge (Kingston Hospital NHS Foundation Trust, Kingston-upon-Thames, UK); Gulsum Anderson (University of Pittsburgh Medical Centre, Pittsburgh, USA); Vu Dinh (Mel Mays, University of Pittsburgh Medical Centre, Pittsburgh, USA); Anna Phillips (University of Pittsburgh Medical Centre, Pittsburgh, USA); Dhiraj Yadav (University of Pittsburgh Medical Centre, Pittsburgh, USA)

## Supplementary Material

znaf155_Supplementary_Data

## Data Availability

The data that support the findings of this study are available from the corresponding author upon reasonable request.
