# Supplementary material for: Pain intensity and prognosis of acute pancreatitis in an international, prospective study
Source: Br J Surg. 2025 Aug 19;112(8):znaf155. doi: 10.1093/bjs/znaf155 (PMC12362069; doi:10.1093/bjs/znaf155)
Supplement: znaf155_Supplementary_Data [file znaf155_supplementary_data.zip › Supplementary_material.docx]

**Pain intensity and prognosis of acute pancreatitis in an international, prospective study**

Cecilie Siggaard Knoph, PhD^1,2^, PAINAP Collaborative*, Sanjay Pandanaboyana, FRCS^3,4^

^1^ Centre for Pancreatic Diseases, Department of Gastroenterology & Hepatology, Aalborg University Hospital, Aalborg, Denmark.

^2^ Department of Clinical Medicine, Aalborg University, Aalborg, Denmark.

^3^ HPB and Transplant Unit, Freeman Hospital, Newcastle upon Tyne, UK.

^4^ Population Health Sciences Institute, Newcastle University, Newcastle Upon Tyne, UK.

* PAINAP collaborative authors are listed in the Supplementary File.

**Corresponding author.** Professor Sanjay Pandanaboyana, FRCS, MPhil, Consultant HPB and Transplant Surgeon; HBP and Transplant Unit, Freeman Hospital, Freeman Road, Newcastle Upon Tyne, Email: sanjay.pandanaboyana@ncl.ac.uk; Phone: 0044 0191244800

ORCID ID: 0000-0003-3099-2197

Twitter: @sanjay_HPB

**Supplementary Materials - Index**

| **Supplementary Appendixes** |  |
| --- | --- |
|  |  |
| **Supplementary Figures and Tables (Supl file 2)** |  |
| Table S1 | *page 10* |
| Table S2 | *page 11* |
| Table S3 | *page 12* |
| Table S4 | *page 13* |
| Table S5 | *page 14* |

**Supplementary File 2**

**Supplementary Figures and Tables**

**Table S1:** Outcome details stratified according to pain intensity.

|  | No/mild pain,  NRS 0-3  (n = 183) | Moderate pain,  NRS 4-6  (n = 503) | Severe pain,  NRS 7-10  (n = 1,041) | P-value | Missing data,  n (%) |
| --- | --- | --- | --- | --- | --- |
| AP severity, n (%)* |  |  |  | 0.002 | 0 (0) |
| - Mild AP | 139 (76) | 388 (77) | 726 (70) |  |  |
| - Moderately severe AP | 35 (19) | 87 (17) | 205 (20) |  |  |
| - Severe AP | 9 (5) | 28 (6) | 110 (10) |  |  |
|  |  |  |  |  |  |
| Organ failure and type,  n (%) |  |  |  | <0.001 | 0 (0) |
| - No | 158 (88) | 460 (93) | 877 (85) |  |  |
| - Yes** | 21 (12) | 36 (7) | 152 (15) |  |  |
| - Respiratory | 16 (9) | 30 (6) | 104 (10) | 0.028 |  |
| - Cardiac | 3 (2) | 7 (1) | 33 (3) | 0.082 |  |
| - Renal | 8 (4) | 14 (3) | 92 (9) | <0.001 |  |
|  |  |  |  |  |  |
| Necrosis, n (%)*** |  |  |  | 0.019 | 0 (0) |
| - No | 170 (93) | 441 (88) | 892 (86) |  |  |
| - Yes | 13 (7) | 62 (12) | 149 (14) |  |  |
|  |  |  |  |  |  |
| Fluid collections,  n (%)**** |  |  |  | 0.028 | 0 (0) |
| - No | 157 (86) | 458 (91) | 903 (87) |  |  |
| - Yes | 26 (14) | 45 (9) | 138 (13) |  |  |
|  |  |  |  |  |  |
| Median length of admission, days (IQR) | 6 (4-11) | 6 (4-10) | 7 (4-12) | 0.003 | 18 (10) |
|  |  |  |  |  |  |
| 30-day Mortality, n (%) |  |  |  | 0.210 | 15 (9) |
| - No | 178 (99) | 486 (98) | 999 (97) |  |  |
| - Yes | 2 (1) | 12 (2) | 35 (3) |  |  |

**Notes:** *According to revised Atlanta criteria, **A subset of patients has multiorgan failure and are counted under several organ failure types, ***Including acute necrotic collections and walled-off necrosis, ****Including acute fluid collections and pseudocysts.

**Table S2:** Multivariable analyses of pain intensity and the odds of developing moderately severe or severe acute pancreatitis, organ failure (any, respiratory, or renal), necrosis, or fluid collections in a subgroup of patients with pain ≤24 hours prior to admission (n =900).

|  | **Adjusted Odds ratios**  **(95 % confidence interval)*** | **P-value** |
| --- | --- | --- |
| **Moderately severe/severe AP** |  |  |
| Moderate pain (vs. no/mild pain) | 1.04 (0.52-2.05) | 0.916 |
| Severe pain (vs. no/mild pain) | 1.73 (0.92-3.27) | 0.091 |
|  |  |  |
| **Organ failure** |  |  |
| Moderate pain (vs. no/mild pain) | 0.68 (0.25-1.81) | 0.436 |
| Severe pain (vs. no/mild pain) | 1.72 (0.73-4.07) | 0.215 |
|  |  |  |
| **Respiratory failure** |  |  |
| Moderate pain (vs. no/mild pain) | 1.06 (0.32-3.53) | 0.930 |
| Severe pain (vs. no/mild pain) | 2.12 (0.70-6.36) | 0.182 |
|  |  |  |
| **Renal failure** |  |  |
| Moderate pain (vs. no/mild pain) | 0.76 (0.21-2.68) | 0.667 |
| Severe pain (vs. no/mild pain) | 2.00 (0.66-6.08) | 0.221 |
|  |  |  |
| **Pancreatic necrosis** |  |  |
| Moderate pain (vs. no/mild pain) | 2.32 (0.51-10.56) | 0.278 |
| Severe pain (vs. no/mild pain) | 2.91 (0.66-12.71) | 0.156 |
|  |  |  |
| **Fluid collections** |  |  |
| Moderate pain (vs. no/mild pain) | 0.54 (0.22-1.32) | 0.178 |
| Severe pain (vs. no/mild pain) | 1.04 (0.47-2.32) | 0.920 |

**Notes:** *Adjusted for age, continent, biliary and alcoholic aetiology, Charlson Comorbidity Indexes, and the use of analgesia pre-admission.

**Table S3:** Diagnostic performance of severe baseline pain (NRS 7-10) in diagnosing AP outcomes in a subgroup of patients with pain ≤24 hours prior to admission (n=928).

| **Outcome** | **AUC-ROC** | **Sensitivity** | **Specificity** | **PPV** | **NPV** |
| --- | --- | --- | --- | --- | --- |
| AP severity* | 0.54 (0.50-0.58) | 67 (60-74) | 41 (38-45) | 22 (19-26) | 84 (80-87) |
|  |  |  |  |  |  |
| Organ failure | 0.57 (0.52-0.62) | 73 (62-83) | 41 (38-44) | 10 (8-13) | 95 (92-97) |
|  |  |  |  |  |  |
| Respiratory organ failure | 0.56 (0.50-0.62) | 72 (58-83) | 40 (37-44) | 7 (5-9) | 96 (93-98) |
|  |  |  |  |  |  |
| Renal organ failure | 0.57 (0.51-0.64) | 75 (60-86) | 40 (37-44) | 6 (4-9) | 97 (94-98) |
|  |  |  |  |  |  |
| Necrosis | 0.54 (0.47-0.60) | 67 (53-79) | 40 (37-43) | 7 (5-9) | 95 (92-97) |
|  |  |  |  |  |  |
| Fluid collections | 0.54 (0.48-0.59) | 68 (56-78) | 40 (37-44) | 10 (7-12) | 93 (90-95) |

**Notes:** *according to RAC. Parentheses indicate 95% confidence intervals. AUC-ROC = Area under the curve of the receiver operating characteristics, PPV = Positive predictive value, NPV = Negative predictive value.

**Table S4:** Multivariable analyses of pain intensity and the odds of developing moderately severe or severe acute pancreatitis, organ failure (any, respiratory, or renal), necrosis, or fluid collections in a subgroup of patients with pain >24 hours prior to admission (n =790).

|  | **Adjusted Odds ratios**  **(95 % confidence interval)*** | **P-value** |
| --- | --- | --- |
| **Moderately severe/severe AP** |  |  |
| Moderate pain (vs. no/mild pain) | 0.91 (0.49-1.67) | 0.754 |
| Severe pain (vs. no/mild pain) | 1.37 (0.78-2.42) | 0.272 |
|  |  |  |
| **Organ failure** |  |  |
| Moderate pain (vs. no/mild pain) | 0.62 (0.29-1.33) | 0.218 |
| Severe pain (vs. no/mild pain) | 1.50 (0.76-2.96) | 0.245 |
|  |  |  |
| **Respiratory failure** |  |  |
| Moderate pain (vs. no/mild pain) | 0.66 (0.28-1.54) | 0.335 |
| Severe pain (vs. no/mild pain) | 1.16 (0.54-2.52) | 0.699 |
|  |  |  |
| **Renal failure** |  |  |
| Moderate pain (vs. no/mild pain) | 0.53 (0.15-1.83) | 0.315 |
| Severe pain (vs. no/mild pain) | 2.72 (1.01-7.35) | 0.049 |
|  |  |  |
| **Pancreatic necrosis** |  |  |
| Moderate pain (vs. no/mild pain) | 2.25 (1.00-5.09) | 0.050 |
| Severe pain (vs. no/mild pain) | 2.34 (1.08-5.09) | 0.032 |
|  |  |  |
| **Fluid collections** |  |  |
| Moderate pain (vs. no/mild pain) | 0.58 (0.29-1.15) | 0.118 |
| Severe pain (vs. no/mild pain) | 0.87 (0.47-1.62) | 0.669 |

**Notes:** *Adjusted for age, continent, biliary and alcoholic aetiology, Charlson Comorbidity Indexes and the use of analgesia pre-admission.

**Table S5:** Diagnostic performance of severe baseline pain (NRS 7-10) in diagnosing AP outcomes in a subgroup of patients with pain >24 hours prior to admission (n=816).

| **Outcome** | **AUC-ROC** | **Sensitivity** | **Specificity** | **PPV** | **NPV** |
| --- | --- | --- | --- | --- | --- |
| AP severity* | 0.54 (0.51-0.58) | 66 (60-71) | 43 (38-47) | 39 (35-44) | 69 (64-74) |
|  |  |  |  |  |  |
| Organ failure | 0.57 (0.53-0.61) | 72 (64-79) | 42 (38-46) | 20 (16-24) | 88 (84-92) |
|  |  |  |  |  |  |
| Respiratory organ failure | 0.54 (0.49-0.59) | 68 (58-77) | 41 (37-44) | 13 (11-17) | 90 (87-93) |
|  |  |  |  |  |  |
| Renal organ failure | 0.63 (0.58-0.68) | 84 (73-92) | 42 (38-45) | 12 (9-15) | 97 (94-98) |
|  |  |  |  |  |  |
| Necrosis | 0.54 (0.50-0.58) | 66 (59-73) | 41 (37-45) | 23 (19-27) | 82 (78-86) |
|  |  |  |  |  |  |
| Fluid collections | 0.53 (0.48-0.57) | 65 (57-74) | 41 (37-44) | 17 (14-21) | 86 (82-90) |

**Notes:** *according to RAC. Parentheses indicate 95% confidence intervals. AUC-ROC = Area under the curve of the receiver operating characteristics, PPV = Positive predictive value, NPV = Negative predictive value.
